# Bioassay-Guided Isolation of Neuroprotective Compounds from *Uncaria rhynchophylla* against Beta-Amyloid-Induced Neurotoxicity

**DOI:** 10.1155/2012/802625

**Published:** 2012-06-20

**Authors:** Yan-Fang Xian, Zhi-Xiu Lin, Qing-Qiu Mao, Zhen Hu, Ming Zhao, Chun-Tao Che, Siu-Po Ip

**Affiliations:** ^1^School of Chinese Medicine, The Chinese University of Hong Kong, Shatin, Hong Kong; ^2^Department of Medicinal Chemistry and Pharmacognosy, College of Pharmacy, University of Illinois at Chicago, Chicago IL 60607, USA

## Abstract

*Uncaria rhynchophylla* is a component herb of many Chinese herbal formulae for the treatment of neurodegenerative diseases. Previous study in our laboratory has demonstrated that an ethanol extract of *Uncaria rhynchophylla* ameliorated cognitive deficits in a mouse model of Alzheimer's disease induced by D-galactose. However, the active ingredients of *Uncaria rhynchophylla* responsible for the anti-Alzheimer's disease activity have not been identified. This study aims to identify the active ingredients of *Uncaria rhynchophylla* by a bioassay-guided fractionation approach and explore the acting mechanism of these active ingredients by using a well-established cellular model of Alzheimer's disease, beta-amyloid- (A**β**-) induced neurotoxicity in PC12 cells. The results showed that six alkaloids, namely, corynoxine, corynoxine B, corynoxeine, isorhynchophylline, isocorynoxeine, and rhynchophylline were isolated from the extract of *Uncaria rhynchophylla*. Among them, rhynchophylline and isorhynchophylline significantly decreased A**β**-induced cell death, intracellular calcium overloading, and tau protein hyperphosphorylation in PC12 cells. These results suggest that rhynchophylline and isorhynchophylline are the major active ingredients responsible for the protective action of *Uncaria rhynchophylla* against A**β**-induced neuronal toxicity, and their neuroprotective effect may be mediated, at least in part, by inhibiting intracellular calcium overloading and tau protein hyperphosphorylation.

## 1. Introduction

Alzheimer's disease (AD), a neurodegenerative disorder characterized by a progressive loss of learning, memory, and other cognitive functions, is the most common form of dementia in the elderly. The pathological hallmarks of AD are extracellular senile plaques and intracellular neurofibrillary tangles [1]. It is well known that deposition of *β*-amyloid (A*β*) is a pivotal event in initiating the neuronal degeneration of AD [2]. A*β* aggregates into amyloid fibrils, which have been reported to be neurotoxic *in vitro* [3] and *in vivo*  [4]. In this connection, the toxic effect of A*β* on a cultured neuronal cells can be used as a screening tool for identifying potential therapeutic agents for AD.

Current clinical treatments of AD patients use acetylcholinesterase inhibitors (AChEIs) and antagonists of *N*-methyl-D-aspartate receptors (NMDA) to slow down the progress of the deterioration of AD. However, effective approaches for delaying the progression of AD are yet to be found to date. Thus, searching for safer, better-tolerated, and effective drugs for the treatment of AD remains an important area of drug discovery. 

Traditional Chinese herbal medicine has been practiced in China for thousands of years, and vast experience has been accumulated for using medicinal herbs for clinical treatment of diseases. Thus, Chinese herbal medicine may be a promising source of effective drugs for treating AD. *Uncaria rhynchophylla* has been extensively used in Chinese herbal medicine to relieve headache, dizziness, tremors, and hypertension-induced convulsion [5–7]. In recent years, *Uncaria rhynchophylla* has been  shown to be effective for inhibiting A*β* fibril formation, disassembling performed A*β* fibrils [8] and antiacetylcholinesterase [9]. Previous study in our laboratory demonstrated that an ethanol extract of *Uncaria rhynchophylla* significantly reversed cognitive deficits induced by D-galactose, a mouse model of AD [10]. Phytochemical study has shown that alkaloids, terpenoids, and flavonoids are the major chemical ingredients of *Uncaria rhynchophylla* [7]. However, the bioactive principles responsible for the protective action of *Uncaria rhynchophylla* have not been identified. The present study aims to identify the active constituents of *Uncaria rhynchophylla* using bioassay-guided fractionation. Furthermore, the acting mechanism of these active ingredients is explored by using a well-established cellular model of Alzheimer's disease, beta-amyloid- (A*β*-) induced neurotoxicity in PC12 cells.

## 2. Materials and Methods

### 2.1. Chemicals and Reagents

Nerve growth factor (NGF), 3-(4,5-dimethylthiazol-2-yl)-2,5-diphenyltetrazolium  bromide (MTT), and the fragment of *β*-amyloid peptide (A*β*_25–35_) were purchased from Sigma-Aldrich (St. Louis, MO, USA). Fura 2-AM, Dulbecco's modified Eagle's medium (DMEM), horse serum, fetal bovine serum, and a penicillin/streptomycin mixture were purchased from Gibco-Invitrogen (Grand Island, NY, USA). All other solvents and chemicals used in the study were of analytical grade.

### 2.2. Plant Material

The dried stem with hooks of *Uncaria rhynchophylla* was purchased from Zhixin Pharmaceutical Co., a GMP-certified supplier of Chinese medicinal herbal materials (Guangzhou, China). It was authenticated to be the dried rhizome of *Uncaria rhynchophylla* (Miq.) Miq. ex Havil. by Ms. Y. Y. Zong, School of Chinese Medicine, The Chinese University of Hong Kong, Hong Kong, where a voucher specimen (no. 091220) has been deposited.

### 2.3. Preparation of Aggregated A*β*_25–35_

The aggregated A*β*_25–35_ was prepared according to a method described previously [11]. Briefly, A*β*_25–35_ was dissolved in sterile distilled water at a concentration of 1 mM and incubated at 37°C for 4 days to form the aggregation. It was stored at −20°C until use.

### 2.4. Extraction, Fractionation, Isolation, and Identification

*Uncaria rhynchophylla* (1 kg) was macerated in 6 L of 70% aqueous ethanol for 24 h at room temperature and then refluxed for 30 min. The extraction was repeated twice. The pooled fractions were concentrated using a rotary evaporator at reduced pressure at 40°C to yield 140 g of extract (UR-E). The extract was resuspended in water and then transferred into a separatory funnel. The solution was partitioned with ethyl acetate and 1-butanol successively to obtain the ethyl acetate-soluble fraction (UR-E-EA, 43 g), the 1-butanol-soluble fraction (UR-E-B, 28 g), and the water-soluble fraction (UR-E-W, 67.5 g), respectively. The UR-E-B was further separated by column chromatography on a Diaion HP-20 column eluted with H_2_O-MeOH (100 : 0, 80 : 20, 70 : 30, 60 : 40, 40 : 60, 30 : 70, 20 : 80, 10 : 90, and 0 : 100) and acetone, successively, to yield 10 major fractions (UR-E-B-Fr. 1–10). The fraction eluted by H_2_O/MeOH (30 : 70) (UR-E-B-Fr. 6) was further separated by a semipreparative HPLC column (Alltima C18 column, 10 × 250 mm, 5 *μ*m) and eluted with 0.01 mmol/L triethylamine in 80% (v/v) aqueous methanol at a flow rate of 3 mL/min to obtain four fractions (UR-E-B-Fr. 6–1 to 4). UR-E-B-Fr. 6–2 was then separated by a semipreparative HPLC column and eluted with 0.01 mmol/L triethylamine in 70% (v/v) aqueous methanol at a flow rate of 2 mL/min to obtain corynoxine (20 mg) and corynoxine B (20 mg). Corynoxeine (63 mg), isorhynchophylline (50 mg), isocorynoxeine (138 mg), and rhynchophylline (100 mg) were purified from UR-E-B-Fr. 6–3 using the semipreparative HPLC column under the following condition: mobile phase, 0.01 mmol/L triethylamine in 70% (v/v) aqueous methanol; flow rate, 3.0 mL/min.

The structures of corynoxine, corynoxine B, corynoxeine, isorhynchophylline, isocorynoxeine, and rhynchophylline were identified by comparing their ^1^H, ^13^C NMR spectroscopic data (Bruker NMR spectrometer, 400 MHz) with published data [12–16].

### 2.5. Cell Culture and Drug Treatment

Rat pheochromocytoma cells (PC12 cells) were obtained from the American Type Culture Collection (Rockville, MD, USA) and cultured in DMEM medium supplemented with penicillin (100 unit/mL), streptomycin (100 *μ*g/mL), 6% fetal bovine serum, and 6% horse serum at 37°C in humidified atmosphere of 95% air and 5% CO_2_. PC12 cells were seeded on poly-D-lysine-coated 96 wells (Coring Incorporated, USA) at a density of 2 × 10^4^ cells/well and allowed to adhere for 24 h at 37°C with the culture medium. PC12 cells were differentiated with 50 ng/mL NGF in serum-free DMEM for 3 days [17]. Thereafter, the culture medium was replaced by fresh serum-free DMEM (without NGF) with or without different concentrations of drugs for 2 h. Then 20 *μ*M of A*β*_25–35_ was added to the cells and incubated for another 24 h. The extracts and isolated compounds were reconstituted in DMSO to produce respective stock solutions and then diluted with culture medium to various concentrations for cell culture experiments. The final DMSO concentration in each sample was less than 0.1%.

### 2.6. Cell Viability Assay

Cell viability was measured by MTT method as described previously [11]. Briefly, after drug treatment, 20 *μ*L of MTT solution (final concentration, 1 mg/mL) was added into each well, and the cells were incubated at 37°C for 4 h. The culture medium was removed, and the formazan crystals were dissolved with 150 *μ*L of DMSO. The optical density of each well was measured using a microplate reader (FLUOstar OPTIMA, BMG Labtech, Germany) at 570 nm. Cell viability was expressed as percentage of nontreated control.

### 2.7. Measurement of Intracellular Calcium Concentration

The concentration of intracellular calcium was determined by a method described previously [18]. Briefly, PC12 cells were differentiated with NGF for 3 days. The cells were pretreated with rhynchophylline (100 *μ*M) or rhynchophylline (100 *μ*M) for 2 hours and then treated with 20 *μ*M of A*β*_25–35_ for 24 hours. At the end of the treatment, the cells were collected and incubated with the culture medium containing 5 *μ*M Fura-2/AM at 37°C for 50 min. Subsequently, the cells were washed twice with HBSS and resuspended in HBSS solution containing 0.2% bovine serum albumin. The intracellular calcium concentration was determined by setting excitation wavelengths at 340 nm and 380 nm; emission wavelength at 510 nm, using a fluorescence spectrophotometer (Shimadzu, RF-5301, Japan). The concentration of intracellular calcium was expressed as percentage of nontreated control.

### 2.8. Western Blotting Analysis

The PC12 cells were seeded onto 100 mm^2^ dish at 5 × 1^6^ cells/dish. The cells were washed twice with D-Hanks solution after drug treatment. The cells were harvested and lysed with lysis buffer. Protein samples were separated by SDS-PAGE for 2 h at 80 V. The separated proteins were transferred to PVD membranes using a transblotting apparatus (Bio-Rad Laboratories, USA) for 30 min at 15 V. The membranes were blocked with 5% (w/v) nonfat milk in TBS-T (Tris-buffer saline containing 0.1% Tween-20) at room temperature for 2 h and subsequently incubated at 4°C overnight with appropriate amount of primary antibody against Tau, p-Tau (Ser 396), p-Tau (Ser 404), p-Tau (Thr 205), and *β*-actin (Santa Cruz Biotechnology Inc., USA). Then the membrane was washed with TBS-T for three times, and probed with horseradish peroxidase-conjugated secondary antibody at room temperature for 1 h. To verify equal loading of samples, the membranes were incubated with monoclonal antibody *β*-actin, followed by a horseradish peroxidase-conjugated goat anti-mouse IgG. The membrane again was washed with TBS-T for three times and finally, the protein bands were visualized by the ECL western blotting detection reagents (Amersham Biosciences, Buckinghamshire, UK). The intensity of each band was analyzed using Image J software (NIH Image, Bethesda, MD, USA).

### 2.9. Statistical Analysis

Data were expressed as mean ± SEM. Multiple group comparisons were performed using one-way analysis of variance (ANOVA) followed by Dunnett's test to detect intergroup differences. GraphPad Prism software was used to perform the statistical analysis (Version 4.0; GraphPad Software Inc., San Diego, CA). A difference was considered statistically significant if the *P* value was less than 0.05.

## 3. Results

### 3.1. Isolation and Structural Determination of the Isolated Compounds

**Figure 1:**
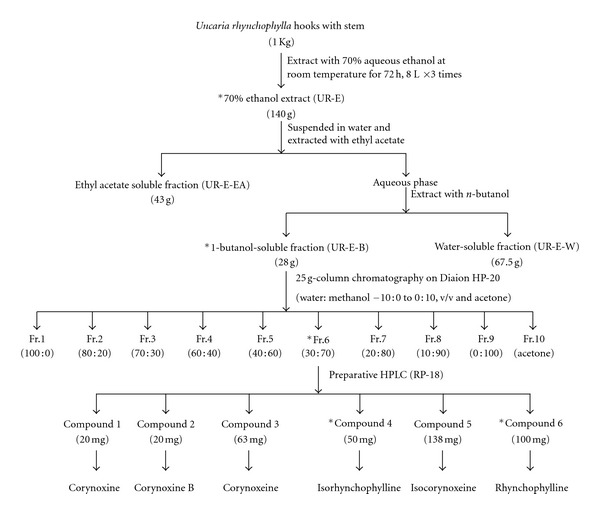
Extraction scheme for the isolation of the six alkaloids from *Uncaria rhynchophylla*. * Biologically active fractions or compounds.

**Figure 2:**
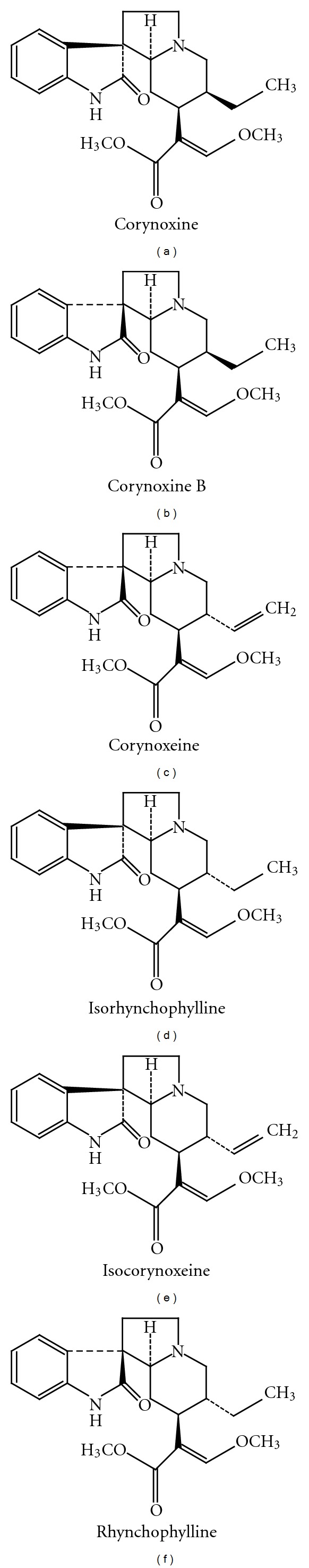
Chemical structures of the six alkaloids isolated from *Uncaria rhynchophylla. *

Figure 1 schematically depicted the extraction procedure leading to the isolation of the pure compounds. The structures of these compounds were identified as corynoxine, corynoxine B, corynoxeine, isorhynchophylline, isocorynoxeine, and rhynchophylline, respectively, based on the detailed interpretation of their ^1^H, ^13^C NMR spectroscopic data. The chemical structures of these isolated alkaloid compounds were shown in Figure 2.

### 3.2. Effect of Different Fractions and Isolated Compounds on A*β*_25–35_-Induced Cells Death in PC12

**Figure 3:**
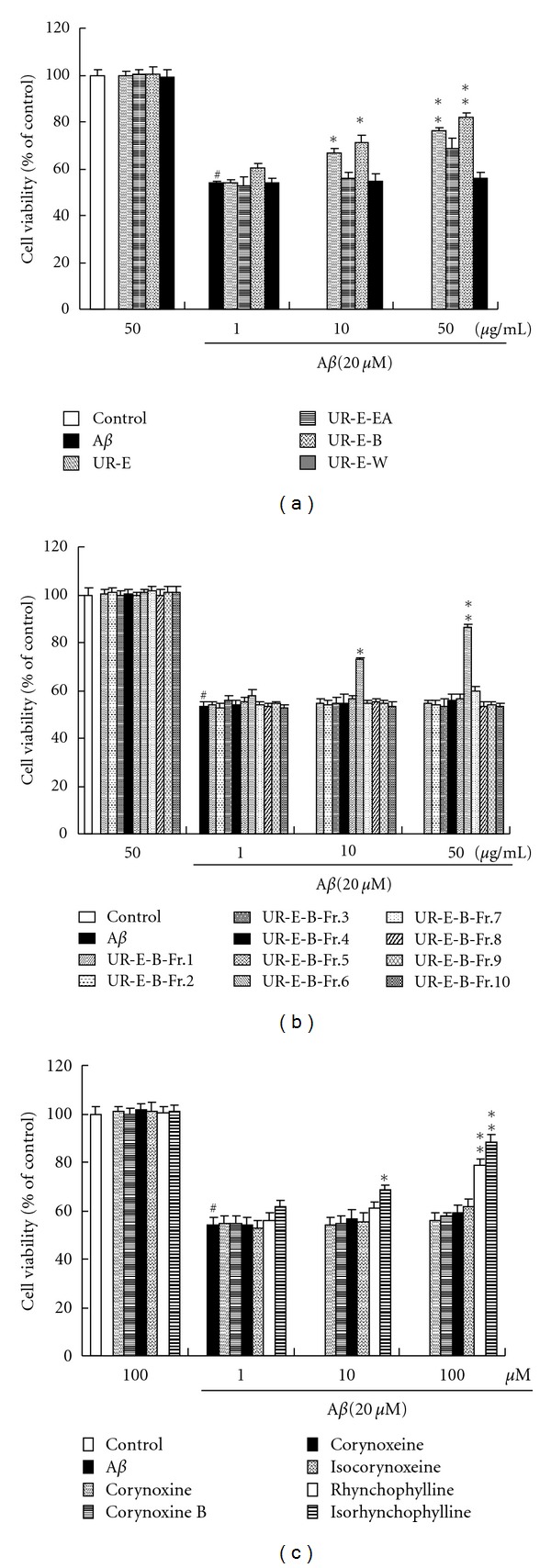
Effect of different extracts (a), fractions (b), and isolated compounds (c) from *Uncaria rhynchophylla* on cell viability in A*β*_25–35_-treated PC12 cells. Values given are the mean ± SEM (*n* = 6). ^#^ *P* < 0.01 compared with the control group; *P < 0.05 and **P < 0.01 compared with the A*β*_25–35_-treated control.

 As shown in Figures 3(a) and 3(b), treating the cells with 20 *μ*M of A*β*_25–35_ for 24 h caused a significant decrease in cell viability (54% of the control). Treating PC12 cells with different fractions (50 *μ*g/mL) or isolated compounds (100 *μ*M) from *Uncaria rhynchophylla *had no effect on cell viability as compared to the control group (Figures 3(a)–3(c)). Pretreatment of the cells with UR-E, UR-E-B, and UR-E-B-Fr.6 (10 and 50 *μ*g/mL) significantly increased cell viability when compared with A*β*_25–35_-treated control, while other fractions from *Uncaria rhynchophylla* had no effect on cell viability in A*β*_25–35_-treated PC12 cells. UR-E-B fraction elicited more effective protection against A*β*_25–35_-induced cell death in PC12 cells when compared with UR-E, UR-E-EA, and UR-E-W fractions. Among these isolated compounds, only rhynchophylline and isorhynchophylline significantly increased the cell viability in A*β*_25–35_-treated PC12 cells (Figure 3(c)), suggesting that rhynchophylline and isorhynchophylline may be the key active components of  *Uncaria* *rhynchophylla*.

### 3.3. Effect of Rhynchophylline and Isorhynchophylline on Intracellular Calcium Concentration in A*β*_25–35_-Treated PC12 Cells

**Figure 4:**
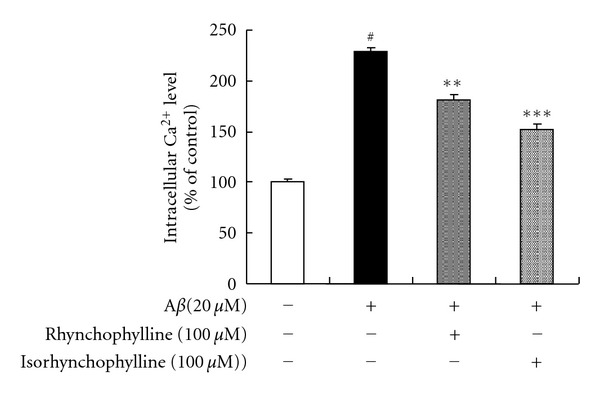
Effect of rhynchophylline and isorhynchophylline on intracellular calcium level in A*β*_25–35_-treated PC12 cells. Values given are the mean ± SEM (*n* = 6). ^#^ *P* < 0.01 compared with the control group; *P < 0.05 and **P < 0.01 compared with the A*β*_25–35_-treated control.

As shown in Figure 4, treating PC12 cells with 20 *μ*M A*β*_25–35_ for 24 h caused a significant increase in the intracellular calcium level (230% of the control), while pretreating the cells with rhynchophylline and isorhynchophylline (100 *μ*M) significantly decreased the intracellular calcium level in A*β*_25–35_-treated PC12 cells.

### 3.4. Effect of Rhynchophylline and Isorhynchophylline on Tau Hyperphosphorylation in *Aβ*_25–35_-Treated PC12 Cells

**Figure 5:**
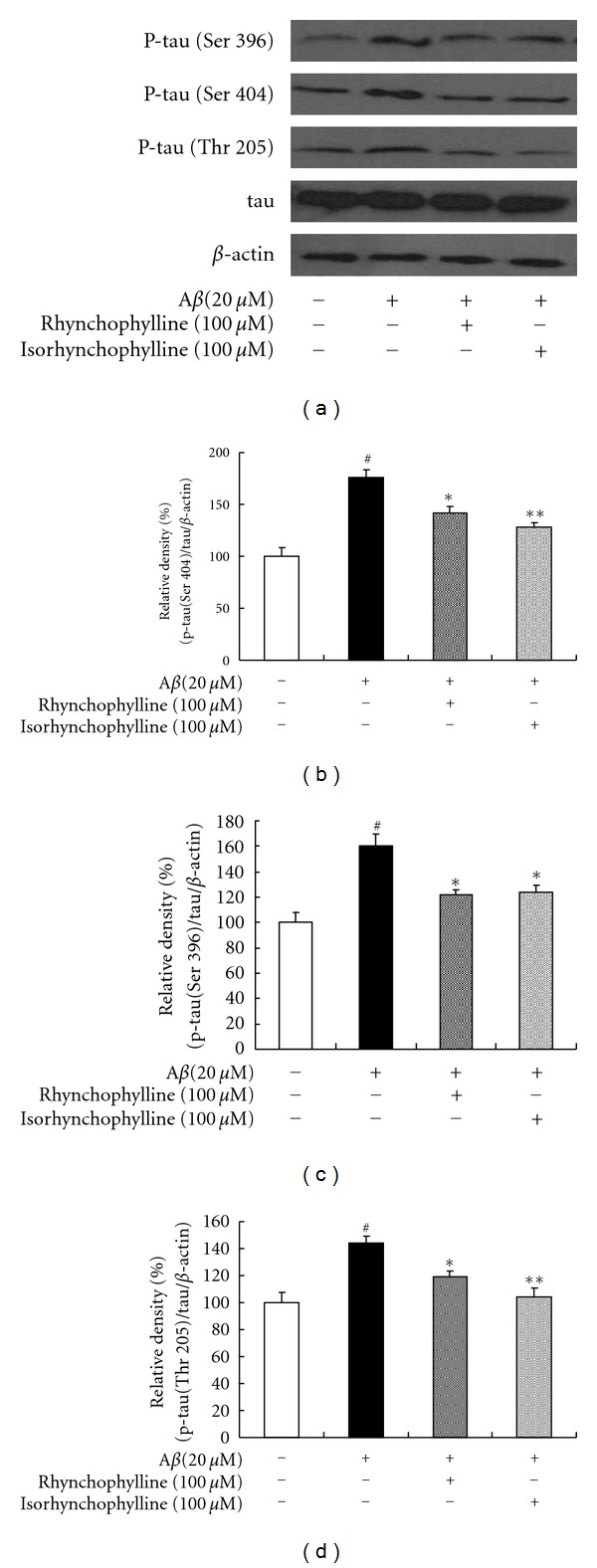
Effect of rhynchophylline and isorhynchophylline on tau protein hyperphosphorylation in A*β*_25–35_-treated PC12 cells. The tau protein hyperphosphorylation was assessed by measuring the phosphorylated tau protein (at Thr 205, Ser 396, and Ser 404 sites) and total tau. Values given are the mean ± SEM (*n* = 3). ^#^ *P* < 0.01 compared with the control group; *P < 0.05 and **P < 0.01 compared with the A*β*_25–35_-treated control.

 As shown in Figure 5, tau protein hyperphosphorylation at Thr 205, Ser 396, and Ser 404 sites was significantly increased (144%, 160%, and 176% of the control, resp.) when treating the cells with 20 *μ*M  A*β*_25–35_ for 24 h. However, phosphorylation of tau protein was significantly inhibited by pretreating the cells with rhynchophylline and isorhynchophylline (100 *μ*M) for 2 h. Meanwhile, the total tau protein did not change significantly for all treatments.

## 4. Discussion

In Chinese herbal medicine, *Uncaria rhynchophylla* is classified as a liver-pacifying and wind-extinguishing herb and is commonly used for treating central nervous system-related symptoms such as tremor, seizure, and epilepsy [19]. Although the neuroprotective effect of *Uncaria rhynchophylla *has been well studied [20–22], this is the first evidence to report for the identification of active anti-AD ingredients from *Uncaria rhynchophylla* by using the bioassay-guided fractionation approach. Six alkaloid compounds including corynoxine, corynoxine B, corynoxeine, isorhynchophylline, isocorynoxeine, and rhynchophylline were isolated and characterized from the *Uncaria rhynchophylla*. Among these compounds, only rhynchophylline and isorhynchophylline, the major tetracyclic oxindole alkaloids present in *Uncaria rhynchophylla* [23], significantly attenuated A*β*_25–35_-induced cell death, the intracellular calcium overload, and tau protein hyperphosphorylation in PC12 cell. Recently, the protective effects of rhynchophylline and isorhynchophylline on different models of neurotoxicity have been described [11, 23, 24]. Based on these findings, rhynchophylline and isorhynchophylline may be the major active ingredients of *Uncaria rhynchophylla*  for its anti-AD activity.

The intracellular calcium concentration plays a critical role in the neuron development. Recent researches have revealed that the neurotoxicity induced by A*β* is mediated by the overloading of intracellular calcium in primary neurons such as hippocampal neurons [25] and cortical neurons [26]. In addition, the accentuation of the intracellular calcium has been considered as one of the activating pathways for A*β*-induced neurotoxicity [27]. Therefore, the blockage of intracellular calcium overloading might provide neuroprotection against A*β*-induced cell death in PC12 cells. Our finding indicated that treating PC12 cells with A*β*_25–35_ significantly increased intracellular calcium levels, whereas pretreating the cells with rhynchophylline and isorhynchophylline was able to inhibit the intracellular calcium influx which may contribute to the neuroprotective effect of rhynchophylline and isorhynchophylline.

Continuous calcium influx can induce the phosphorylation of tau protein [28]. Neurofibrillary tangles are intracellular aggregates of hyperphosphorylated tau protein which is commonly known as a primary pathological hallmark of AD. It has been reported that hyperphosphorylation of tau protein is an essential element for A*β*-induced neurotoxicity [29]. Upon A*β* stimulation, hyperphosphorylation of tau protein is significantly increased at the AD-related epitope and paired helical filament, resulting in a cytoskeletal destabilization, memory dysfunction, and death of the neurons [30, 31]. It has been suggested that tau phosphorylation is the limiting factor in A*β*-induced cell death [32]. In addition, it has been reported that tau hyperphosphorylations is increased significantly in postmortem brain tissues of AD patients [33]. Thus, pharmaceutical agents that can inhibit A*β*-induced tau phosphorylation are potential candidates for the effective treatment of AD. In this study, A*β* was found to cause a marked elevation of tau protein hyperphosphorylation in PC12 cells, while pretreating the cells with rhynchophylline and isorhynchophylline significantly decreased the level of tau hyperphosphorylation. The results suggest that the inhibition of tau protein hyperphosphorylation rhynchophylline and isorhynchophylline may be one of the acting mechanisms for the protective effect of rhynchophylline and isorhynchophylline against A*β*-induced neurotoxicity.

## 5. Conclusions

 In summary, our results demonstrated that rhynchophylline and isorhynchophylline significantly decreased A*β*_25–35_-induced cell death, calcium overloading, and tau protein hyperphosphorylation in PC12 cells, suggesting that rhynchophylline and isorhynchophylline may be the major active ingredients of *Uncaria  rhynchophylla*  for the treatment of AD, and their neuroprotective effect may be mediated, at least in part, by inhibition of intracellular calcium overloading and tau protein hyperphosphorylation. Further investigation on the potential use of rhynchophylline and isorhynchophylline in animal model of AD is warranted.
